# Integrating Clinical and Transcriptomic Profiles Associated with Vitamin D to Enhance Disease-Free Survival in Cervical Cancer Recurrence Using the CatBoost Algorithm

**DOI:** 10.3390/diagnostics15131579

**Published:** 2025-06-21

**Authors:** Geeitha Senthilkumar, Renuka Pitchaimuthu, Seshathiri Dhanasekaran, Prabu Sankar Panneerselvam

**Affiliations:** 1 Department of Information Technology, M. Kumarasamy College of Engineering, Thalavapalayam, Karur 639113, Tamil Nadu, India; geethas.it@mkce.ac.in (G.S.); renukap.it@mkce.ac.in (R.P.); 2 Department of Computer Science, UiT The Arctic University of Norway, 9037 Tromsø, Norway; 3 Shanmuga Hospital, Salem 636007, Tamil Nadu, India; drprabusankar@smrft.org

**Keywords:** cervical cancer recurrence, vitamin D, disease-free survival, machine learning predictors, long non-coding RNA

## Abstract

**Background/Objectives**: Cervical cancer is a leading cancer-related cause of death among women, with recurrence being a serious clinical issue. Recent evidence demonstrates that long non-coding RNAs (lncRNAs) affect cancer recurrence. This research investigates vitamin D’s regulatory actions in the recurrence of cervical cancer, centering on the involvement of lncRNA. Clinical data on 738 patients shows that greater serum vitamin D levels are linked to reduced recurrence rates and enhanced disease-free survival (DFS). **Methods**: A transcriptomic analysis of CaSki cervical cancer cells using data from the GEO dataset GSE267715 identified that vitamin D controls genes that prevent cervical cancer recurrence. Machine learning predictors CatBoost, LightGBM, Extra Trees, and Logistic Regression and feature selection methods such as ANOVA F-test, mutual information, Chi-squared test, and Recursive Feature Elimination (RFE) are used to identify predictors of recurrence, evaluating model performance using accuracy, precision, recall, ROC AUC, confusion matrices, and ROC curves. **Result**: CatBoost performs the best overall, producing an accuracy of 95.27%. CatBoost provided an ROC AUC of 0.9930, a precision of 0.9296, and a recall of 0.9706, and this implies a significant trade-off between the ability to detect metastatic cases correctly. **Conclusions**: These data identify the therapeutic potential of vitamin D as a regulatory compound and lncRNA as a potential therapeutic target in the recurrence of cervical cancer.

## 1. Introduction

The fourth most common gynecological disease, cervical carcinoma, contributes significantly to the death rate of women worldwide [1]. An estimated 600,000 instances and 340,000 deaths from cervical cancer occur annually, making it a major worldwide health threat. Cervical cancer is diagnosed at a mean age of 53 and causes death at an average age of 59, which accounts for 8% of cancer-related deaths among women globally [2,3]. One of the leading causes of cervical carcinoma is a virus from the human papillomavirus (HPV). Almost 95 percent of aggressive cervical tumors have HPV DNAs [4]. The key risk variables for cervical carcinoma are immune suppression; smoking; a history of pregnancy; prolonged usage of contraceptive medications; and human papillomavirus (HPV), especially HPV 16 and HPV 18 [5]. According to research, genetic alterations with changed expression of tumor-suppressive genes act in conjunction with the transmission of HPV, which might cause cervical carcinoma to grow autonomously [6].

Individuals with staging IB to IIA face a 10 to 20 percent chance of recurrence, and those with staging IIB to IVA had a 50 to 70 percent chance, according to the findings released through the International Federation of Obstetrics and Gynecology. In addition, the chance of survival is poor for individuals with distant metastases [7]. Cervical cancer screening, diagnosis, and therapy have advanced significantly. Cervical cytology screening and primary hrHPV testing are two important developments that have significantly reduced the death rate of cervical cancer in people between the ages of 21 and 65 [8]. Clinical characteristics offer a valuable point of reference for estimating the likelihood of recurrence in treatable cervical cancer [9]. ML models can concurrently forecast survival and location-specific recurrence, and they may be a more analytically sound method for cervical cancer forecasting than previous models [10]. This research highlights the clinical trials and meta-analyses suggesting a potential benefit of vitamin D supplementation in improving immune surveillance and reducing the risk of relapse in cervical cancers. Long-term vitamin D supplementation significantly improved the histological regression of cervical intraepithelial neoplasia CIN 1 and CIN 2, which led to a positive change in metabolic parameters. Vitamin D appears to have anti-inflammatory and metabolic benefits, which could indirectly support tissue recovery and immune function in HPV-related neoplasia [11,12,13].

The nine-lncRNA signature showed greater prediction precision than the FIGO stage. Additionally, the stratified analysis showed that the nine-lncRNA signature forecasts cervical cancer progression within the FIGO stage [14]. The clinical and molecular results further demonstrate how HPV infection and integration play a significant role in causing genetic mutations that underlie cervical carcinoma and the stages in cohorts of various racial and geographic backgrounds [15]. Immunosuppressive surroundings are also created by angiogenesis, the spread of cancer, and the carcinoma proliferation of cells. These components are linked to adverse medical outcomes and tumor development [16,17]. Nonetheless, the prognosis for cervical cancer patients is still not good, particularly for individuals who have recurrence or metastatic disease [18]. Significant work has been carried out up to this point to improve chemo-radiotherapy sensitivity and anticipate how patients with CESC will react to radiation. For example, patients with locally advanced cervical cancer have seen increases in CR and LRC rates of 10.2 and 8.4 percent, respectively, when receiving concomitant radiation and cisplatin-based chemotherapy. A few patients do not obtain a sufficient LRC or CR while receiving chemotherapy and radiation treatment concurrently; instead, they have tumor recurrence or metastasis [19]. Radiotherapy performance is predicted by clinical parameters like stages, human papillomavirus (HPV), histological and biomolecular indicators like DNA methylation, hypoxia, tumor microenvironment (TME), cancer stem cells, microRNAs, and lncRNAs [20]. The widespread presence of vitamin D insufficiency has become a significant health problem, prompting worries about its possible links to chronic HPV infection and the advancement of cervical carcinoma. In European countries, the frequency of vitamin D (VD) deficiency varies from 6.9% to 81.8%, while in Asian countries, it varies from 2.0% to 87.5%. Over fifty percent of adults in over half of the countries have a vitamin D deficiency [21].

## 2. Related Works

Vitamin D might have a suppressive impact on cervical cancer because of its possible link to HPV infection. Vitamin D activates genes and mechanisms that play a part in the immune mechanism and are engaged in adaptive and innate immunity [22]. In conclusion, Vitamin D shows promise as a critical variable in the advancement and increase in cervical cancer, which may lower the risk of developing the cancer [23]. A sufficient amount of vitamin D could decrease the chance of cervical cancer in women, as the clinical and preclinical research evaluated here supports a protective role of vitamin D in avoiding HPV-dependent lesions in the cervical cavity and affecting the ongoing progression of cervical carcinoma [24]. Diet and nutrition are significant factors of the cancer prevention and anti-HPV infection strategies seen in cervical cancer. Antioxidants are the primary factors that reduce cervical cancer, and they include vitamins A, C, D, and E. These antioxidants could affect the course of diseases linked to HPV infection differently [25]. The current research states that the duration of recurrence and risk factors are linked with recurrence, time rates of recurrence, and longevity after recurrence among individuals with cervical carcinoma following the initial treatment [26]. In most cervical carcinoma patients, recurrence may be identified early using an accurate evaluation of clinical symptoms. The likelihood of survival results did not correlate with particular diagnostic techniques for detecting recurrence [27]. Long-standing research history and expertise have attempted to discover important risk factors for recurrence because the risk variables are broadly defined [28]. Having a respectable accuracy for prediction [c-index/AUC/R2 > 0.7], forecasting algorithms for cervical cancer toxicity, regional or distant recurrence, and lifespan show encouraging findings [29]. In addition to having significant diagnostic utility, the investigation of particular lncRNAs as modulators of gene expression implicated in pathways of the advancement of cervical cancer also has therapeutic implications for individuals with the disease [30]. lncRNAs are desirable biomarkers for detecting and predicting cervical cancer [31]. Bioinformatics techniques were employed to evaluate the possible contribution of the essential lncRNAs to CC recurrence. Through in vitro investigations, the impact of key lncRNAs on the CC phenotype is ascertained [32]. Following initial therapy, individuals with advanced cervical cancer (CC) have a dismal outcome and no biomarkers to identify those who are more likely to experience a recurrence of CC [33].

## 3. Materials and Methods

The primary research objective is to find the association between vitamin D levels and cervical cancer recurrence and examine vitamin D’s mechanistic role in modulating gene expression lncRNAs that influence recurrence pathways. The dataset supports a statistically significant correlation between higher vitamin D levels and improved disease-free survival (DFS). The dataset has 738 cervical cancer patients’ details and 32 features, including in-depth clinical and demographic data collected from Shanmugha Hospital Salem from 2023 to 2025; their FIGO stages of cancer, from the early to late stages; and 11 various treatment methods, ranging from immunotherapy to radical trachelectomy to chemotherapy. Amongst a total of patients, 100 were found to have a disease recurrence, whereas 638 were free from recurrence during follow-up.

**Figure 1:**
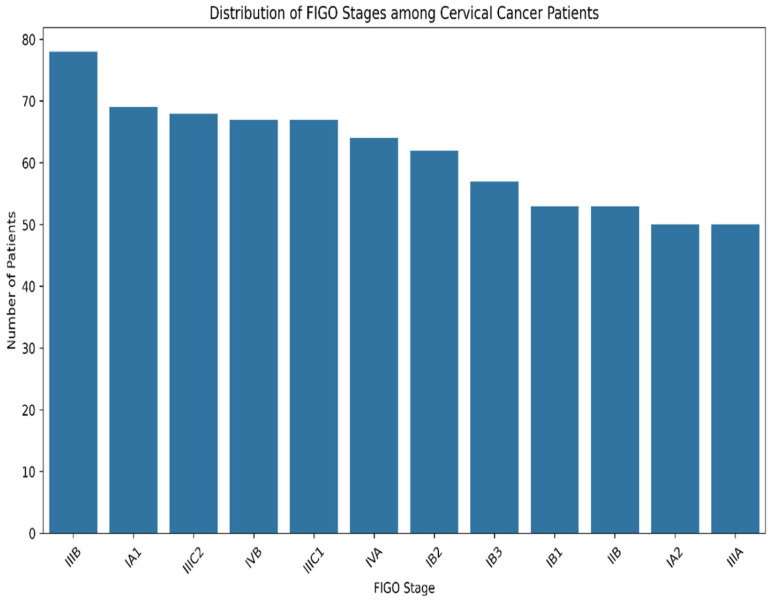
Distribution of FIGO stages among cervical cancer patients.

The dataset also groups patients by five main symptom categories, i.e., abnormal bleeding, discharge, and pain during sex, providing significant information on presenting symptoms and possible correlations with cancer advancement. Figure 1 shows a detailed distribution of FIGO stages among cervical cancer patients. FIGO stages have also been portrayed graphically in an analysis to understand cancer dissemination patterns and intensity levels. Patients with cancer frequently have comorbidities [34]. A detailed symptomatology analysis and comorbid evaluation identify that there are definite clinical presentation types that have significantly higher rates of recurrence in cervical cancer patients.

**Figure 2:**
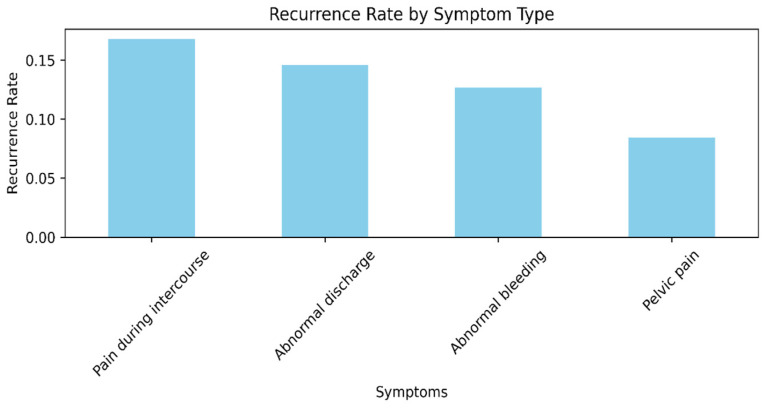
Recurrence rate by symptom type.

Figure 2 shows the recurrence rate by symptom type. Particularly, patients presenting with pain during intercourse and abnormal bleeding are found to have the highest rates of recurrence, meaning these symptoms may be predictive of more advanced or more virulent disease at presentation. Asymptomatic individuals or individuals who present nonspecifically have the lowest recurrence rates, indicating early detection or lower-grade disease [35].

Table 1 describes the clinical presentation of 738 patients who developed cervical cancer. The average serum vitamin D level was 24.84 ng/mL, reflecting widespread deficiency. The average symptom duration was between 6 months and 1 year. The average FIGO stage was 4.26, reflecting a dominance of cancer stages that are moderate to advanced. Lymph node metastasis was identified in 46% of patients, and 13.5% were found to have cancer recurrence. The above baseline data reflect the clinical severity of the group and identify vitamin D deficiency, advanced stage, and metastasis as essential factors in further predictive modeling and biological interpretation.

The nutritional status, specifically levels of vitamin D, were also significant. Patients with higher serum levels of vitamin D were found to have statistically more prolonged disease-free survival (DFS) and lower rates of recurrence, suggesting a potential antiproliferative action of vitamin D in neoplasia of the cervix [36]. Diet quality was also found to be a significant prognosticator. Patients on a high-quality diet yielded the most favorable DFS and rates of lowest recurrence. In contrast, those with low to moderate dietary patterns yielded poorer survival rates. These observations imply incorporating nutritional optimization and supplementation modalities as adjunct treatments for cervix cancer. Cervical cancer survivors frequently engage in unhealthy habits that could lead to early death or disease recurrence [37].

**Figure 3:**
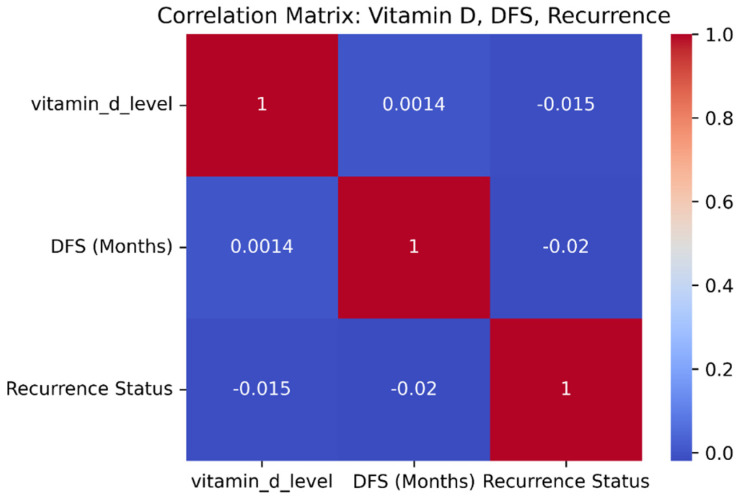
Correlation matrix.

The comparison of nutritional and supplementary factors demonstrates strong correlations with cervical cancer prognostication. An increased level of vitamin D has a positive correlation with DFS and a negative correlation with recurrence, implying that favorable vitamin D levels have a protective response against cancer relapse. Figure 3 is the correlation matrix of vitamin D, disease-free survival, and recurrence status. Correspondingly, dietary habits also make a substantial contribution. High-quality diet patients have the longest DFS rates and lowest recurrence rates. Patients with cervical carcinoma may benefit from specific dietary supplements like probiotics, omega-3 fatty acids, zinc, vitamin D, and folate [38]. On the contrary, those consuming a low- or medium-grade diet have shorter DFS rates and a higher recurrence rate. These findings demonstrate the utility of implementing vitamin D supplementation, along with increased dietary interventions, follow-up treatment, and preventive programs for patients with cervical cancer.

**Figure 4:**
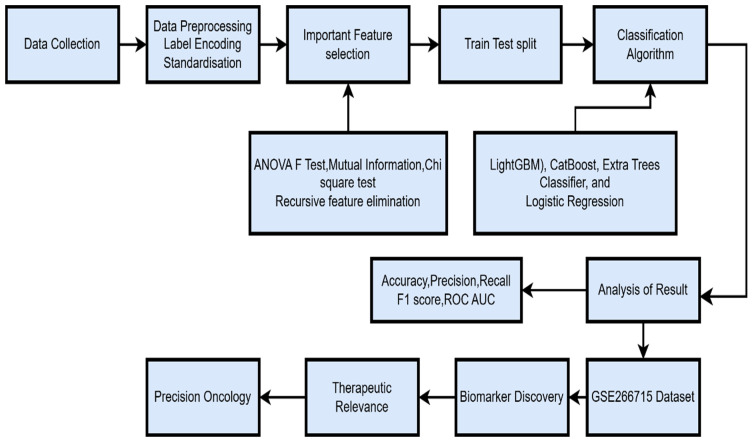
Proposed model architecture.

Figure 4 shows the proposed model architecture—clinical data collected from Shanmugha Hospital, Salem. The data underwent preprocessing, followed by the application of four feature selection techniques. The dataset was split into 80% for training and 20% for testing. Model performance was evaluated using accuracy, precision, recall, F1-score, and ROC AUC metrics. Finally, clinical and demographic data was integrated with the GSE267715 transcriptomic dataset to perform a combined analysis that links vitamin D regulation with lncRNA-mediated recurrence risk in cervical cancer.

### 3.1. Data Preprocessing

The cervical cancer dataset included numerical variables and categorical variables, some of which were missing data points and textual data. We applied a standardized preprocessing pipeline as follows:

#### 3.1.1. Handling Categorical Data: Label Encoding

Preprocessing was conducted on the dataset before model preparation to make it free from quality errors, machine learning algorithm-friendly, and clinically understandable. The dataset was initially converted using label encoding for variables such as symptoms, comorbidities, treatment type, diet patterns, addictive habits, and FIGO stages [39].

$Let\;C=\{C_{1},C_{2}\ldots C_{n}\}$ by categories

(1) $$L\left(C_{i}\right)=i\;for\;i\in \{0,1,\ldots n-1\}$$

Label encoding gives a numerical identifier to each unique category, enabling numerical requirement data to be input into these algorithms. The column Symptoms, where input was given as “none”, “abnormal bleeding”, or “pain during intercourse”, was replaced by numerical representations such as 0, 1, 2, etc. Label encoding applies to tree-based classifiers such as Random Forests, where numerical encoding magnitude does not affect model performance.

#### 3.1.2. Handling Missing Data

Secondly, to preserve the clinical authenticity of the dataset and minimize the bias inherent in imputation methods, complete-case analysis was conducted for missing data. Rows where there were one or more missing values were not considered in the analysis, i.e., were excluded from analysis [40]. Formally, this means that only samples were kept, such that the dataset upon which model training and evaluation were conducted was both uniform and unbiased.

$$Let\;X=\{x_{1},x_{2}\ldots x_{n}\}$$

(2) $$X_{cleaned}=\{x_{i}\in X|\forall_{J,}x_{ij}\neq NA\}$$

#### 3.1.3. Standardization

Thirdly, standardization is applies to all numerical variables, including age, vitamin D level, and post-menopause duration. Standardization was necessary because features had different scales, which can distort model performance [41]. This process involves transforming a raw feature x into a standardized score z using the formula.

(3) $$Z=\frac{X-\mu }{\sigma }$$

where

(4) $$\mu =\frac{1}{n}\sum x_{i},\sigma =\sqrt{\frac{1}{n}\sum {\left(x_{i}-\mu \right)}^{2}}$$

Finally, the dataset is split into a train set and a test set in a proportion of 80:20. The split replicates real-world scenarios where predictive models are tasked with making predictions from unseen data. The approach allows for an objective performance assessment of predictive models and reduces overfitting risks.

(5) $$D=D_{Train}\cup D_{Test,}D_{Train}\cap D_{Test}=0$$

(6) $$\left|D_{Ttrain}\right|=0.8\left|D\right|$$

(7) $$\left|D_{Test}\right|=0.2\left|D\right|$$

### 3.2. Feature Selection

The confusion matrix shows the classification performance and is not directly used to assess feature correlation. Feature selection methods reduce overfitting, increase computational efficiency, and identify the relevant features. We used four strong feature selection methods, ANOVA F-test, mutual information, Chi-squared test, and Recursive Feature Elimination (RFE), to find the most predictive features for cervical cancer relapse. Feature selection plays a critical role in biomedical machine learning as dimensionality reduction, increased model accuracy, and interpretability are achieved by eliminating irrelevant, redundant data.

#### 3.2.1. ANOVA F-Test

The Analysis of Variance F-test is a statistical screening technique to check whether a numeric attribute significantly differs among recurrence classes [42]. It makes a normality assumption of data, along with equal variances, so it is applied to continuous variables like vitamin_d_level and age.

${\bar{y}}_{k}$ is the average value of features for a dataset belonging to group k.

Let $\bar{y_{k}}$ be the mean of group k and $\bar{y}$ be the global mean.$m_{k}$ is the number of samples in group *k*. *m* is the total number of samples. *k* equal to 0 denotes no lymph node metastasis and *k* equal to 1 denotes lymph node metastasis.

(8) $${\bar{y}}_{k}=\frac{1}{m_{k}}\sum_{i:z_{i}=k}y_{i}$$

(9) $$\bar{y}=\frac{1}{m}\sum_{i=1}^{m}y_{i}$$

The between-group variance

(10) $$SSB=\sum_{k=1}^{K}m_{k}{\left({\bar{y}}_{k}-\bar{y}\right)}^{2}$$

*SSB* is the sum of squares between groups. For a higher *SSB*, there are more different groups and the feature is more predictive. The Within-group variance is defined as

(11) $$SSW=\sum_{k=1}^{K}\sum_{i:z_{i}=k}{\left(y_{i}-{\bar{y}}_{k}\right)}^{2}$$

*SSW* is the sum of squares within groups. If the *SSW* is higher, then the feature is less predictive. The *F* statistic is the ratio of between-group variance to within group variance

(12) $$F=\frac{SSB/(K-1)}{SSW/(m-K)}$$

For higher stands, there is a chance to differentiate between non-recurrence and recurrence. In our data, numerical predictors like post-menopause in years and age were found to have significant differences by ANOVA between recurrence groups. In our research, attributes like vitamin D level and age have high *F*-values, reflecting statistically distinct classes of recurrences.

#### 3.2.2. Mutual Information

Mutual information (MI) quantifies how much information a feature has in common with the target, both linearly and nonlinearly. Unlike ANOVA, it can handle numerical and categorical variables [43].

The uncertainty of *Z* is reduced when we know *X*.

The entropy of target *Z*:

(13) $$H\left(Z\right)=-\sum_{z\in Z}p\left(z\right)\log p(z)$$

Entropy measures the uncertainty of *Z* alone. Where *z* belongs to *Z* represents the possible result of random variable *Z*.

Conditional entropy of *Z* given *Y*:

(14) $$H\left(Z|Y\right)=-\sum_{y\in Y}p(y)\sum_{z\varepsilon Z}p\left(z|y\right)\log p(z|y)$$

Conditional entropy measures the remaining uncertainty in *Z* after knowing *Y*.

Mutual Information:

(15) $$I\left(X;Y\right)=H\left(Y\right)-H(Y|X)$$

MI shows a reduction in uncertainty after *X* is determined. Treatment type, FIGO stage, and comorbidities in research displayed high mutual information with recurrence. Treatment type and FIGO stage emerged as having a high content of information about the likelihood of recurrence through MI.

#### 3.2.3. Chi-Squared Test

The Chi-squared test analyzes statistical independence between the binary recurrence condition and a categorical feature. It suits discrete, coded features like symptoms, addictive habits, and FIGO stage [44] and tests whether feature and target are statistically dependent. Large values of chi^2^ are reported for symptoms and FIGO stage, asserting that these are significant features in classifying recurrence.

Measures whether feature and target are statistically dependent.

Step 1: Observed and expected frequencies.

$$O_{i}\text{—}observed\;frequency$$

$$E_{i}\text{—}Expected\;frequency$$

(16) $$E_{i}=\frac{\left(Row\;total\;of\;i\right)*(column\;total\;of\;i)\;}{Grand\;Total}$$

Step 2: Chi-square statistic.

(17) $$Y^{2}=\sum_{i=1}^{m}\frac{{\left(O_{i}-E_{i}\right)}^{2}}{E_{i}}$$

Deviation between observed and expected frequencies is measured.

(18) Degree of freedom=(r−1)(c−1)

#### 3.2.4. Recursive Feature Elimination (RFE)

RFE is a wrapper technique that iteratively adjusts a model and recursively removes the least significant features until a specified number is left. It considers the model’s performance directly, so it is a candidate for data consisting of strong feature interactions. Finding genes with positive coefficients and focusing preventative measures upon them lowers risk factors for cervical cancer recurrence [45].

The logistic regression model estimates and recursively removes the least significant features from a trained model. In this work, RFE identified vitamin_d_level, treatment type, and age as having a significant influence on predicted recurrence.

The logistic regression model estimates by recursively removing the least important features based on a trained model.

Step 1: Train initial model.

(19) $$\hat{z}=\gamma \left(u_{0}+u_{1}y_{1}+u_{2}y_{2}+\cdots +u_{n}y_{n}\right)$$

(20) $$\gamma \left(y\right)=\frac{1}{1+e^{-y}}\;(sigmoid\;function)$$

Step 2: Rank features.

$$Importance\;of\;y_{i}=|u_{i}|$$

$$\left|u_{i}\right|\text{—}more\;influence$$

Step 3: Iterative elimination.

Step 4: Remove features with the smallest $\left|u_{i}\right|$.

Step 5: Retrain the model. Repeat if desired. This is repeated until the top-k features remain.

RFE selected features like treatment type, age, vitamin D level, and post-menopause as highly influential in predicting recurrence.

### 3.3. Classification Algorithms and Their Role in Predicting Lymph Node Metastasis

We aimed to examine the utility of predictive properties, both clinical and molecular, including vitamin D variables, in identifying lymph node metastasis in patients with cervical cancer by empirically applying and evaluating four distinct competent classification methods. These classification methods, Light Gradient Boosting Machine (LightGBM), CatBoost, Extra Trees Classifier, and logistic regression, are all individually equipped to manage high-dimensional, multitype biomedical data. CatBoost and LightGBM are boosted decision tree algorithms that handle categorical variables well and perform excellently on tabular data with nonlinear relationships. Extra Trees was chosen for its ability to capture complex patterns that are robust enough to be overfitted. Logistic regression is a baseline linear model for interpretability and comparison. Using multiple classifiers allows for a more robust evaluation and ensures that the observed trends are not model-specific. This addition enhances the transparency of the machine learning approach. We wanted to compare the relative accuracy of these methods in the classification of lymph node metastasis (binary: present vs. absent), as well as to determine what features, specifically measures of vitamin D, retained predictive power.

#### 3.3.1. LightGBM

LightGBM grows trees leaf-wise, not level-wise, where it picks a leaf, maximizing delta loss during the split, resulting in lower loss and higher accuracy. We set up LightGBM using class weights to counteract the potential imbalance between metastatic and non-metastatic scenarios in the dataset [46]. When training on 80% of the preprocessed dataset and testing on 20%, LightGBM produced excellent classification accuracy using high ROC AUC and high accuracy scores. Interestingly, we computed feature importance scores from the model after training, where we found that vitamin_d_level is the top predictor of involvement of lymph nodes. These results support our hypothesis that vitamin D status critically influences tumor microenvironment and metastatic potential.

(21) $$LGBM\left(\theta \right)=\sum_{i=1}^{m}h\left(z_{i},F\left(y_{i}\right)\right)+\sum_{k=1}^{K}\beta (f_{k})$$

*h* = binary code entropy loss.

$F\left(y_{i}\right)$—model prediction after *i* boosting rounds.

$\beta (f_{k})$—regularization to avoid over-complex trees.

#### 3.3.2. Split Selection in Trees

At each node, LightGBM calculates the Gain:

(22) $$Gain=\frac{1}{2}\left(\frac{{X_{L}}^{2}}{I_{L}+\lambda }+\frac{{X_{R}}^{2}}{I_{R}+\lambda }-\frac{{{(X}_{L}+X_{R})}^{2}}{I_{L}+I_{R}+\lambda }\right)-\gamma$$

*X* = sum of gradients (errors).

*I* = sum of second derivatives (stability). *λ*, *γ* = regularization parameters.

From the dataset collected, LightGBM finds that splitting patients based on vitamin_d_level < 20 ng/mL produces maximum reduction in loss, separating low vitamin D patients at risk of metastasis. Table 2 shows the important feature score of the LightGBM output, which gives the important features top scores.

CatBoost, a categorical boosting algorithm designed by Yandex, was chosen because of its superior performance in dealing with categorical variables and its resistance to overfitting, especially when applied to biomedical data with a mix of variable types. CatBoost employs an “ordered boosting” strategy that prevents information leaks during training and takes advantage of “ordered target statistics” to encode categorical features, maintaining the purity of the learning signal [47]. Such a feature was especially useful in our data, where features such as type of imaging, type of treatment, and symptom duration were initially in categorical or text form. CatBoost was also set to train using class weighting to reduce the effects of label imbalance. CatBoost performed competitively in a test run, sometimes lifting or even topping LightGBM in terms of recall, making it especially useful in accurate metastatic case detection and a top clinical priority. However, feature importance and the persistent appearance of variables about vitamin D among top-performing features during internal validation imply its independent usefulness across programs.

(23) $$Loss=\sum_{i=1}^{n}\left[P_{i}\log\left({\hat{P}}_{i}\right)+\left(1-P_{i}\right)\log\left(1-{\hat{P}}_{i}\right)\right]$$

#### 3.3.3. Extra Trees Classifier

Extremely Randomized Trees is a non-boosted ensemble model used to see how well a randomized forest of decision trees could predict lymph node metastasis from the same set of features [48]. Unlike the usual Random Forests, Extra Trees adds an extra layer of randomness, not just in picking the features but also in choosing the threshold when splitting nodes. This randomness usually means the model has more variance but less bias, making it a great choice when dealing with noisy data. To keep things fair between metastatic and non-metastatic cases, we set the class_weight to “balanced.” It was particularly good at avoiding false positives, which is crucial when considering whether to recommend extra treatments or avoid putting patients through unnecessary procedures. It did not beat LightGBM or CatBoost in overall balanced accuracy. However, the model noted simplicity, transparency, and resilience, and it still turned out to be a valuable model for comparison. When we looked at its feature importance rankings, vitamin D metrics showed up right at the top, which gave even more strength to our original hypothesis.

#### 3.3.4. Logistic Regression

Logistic regression is a baseline model. Set it up with class_weight = ‘balanced’ to treat both classes fairly, and use L2 regularization to keep overfitting in check. Even though it is a pretty straightforward model, it performed surprisingly well, especially when we looked at precision and F1-score. The most effective ML algorithm classifiers for identifying the important predictors discovered to be a logistic regression [49]. Logistic regression was helpful because it clearly showed the direction and strength of key predictors, like vitamin D levels. It gave us a clean, easy-to-trust model compared to the more complex models and a better sense of how well the data separates linearly. We also teamed it up with Recursive Feature Elimination (RFE), letting the model itself help pick out the most important features step by step. Vitamin D levels kept showing up in the top 10 features. That consistency underlined how much vitamin D independently contributed to the classification task.

Logistic regression—Modeling Recurrence Risk Linearly

(24) $$P\left(Z=1|Y\right)=\frac{1}{1+e^{-(\alpha_{0}+\alpha_{1}y_{1}+\alpha_{2}y_{2}+\cdots \alpha_{n}y_{n})}}$$

*Z* = 1 if metastasis.

*Z* = 0 if otherwise.

*Y* = patient features.

$\alpha_{i}$—learned weight from dataset.

Feature selection and classification worked well, minimizing overfitting and boosting generalizability. We applied four strong feature selection strategies, ANOVA F-test, mutual information, Chi-squared test, and Recursive Feature Elimination, to rank and cross-validate the most meaningful predictors. We compared the feature subsets across methods, and it was encouraging to see a significant overlap among the top-ranked features. This consistency strengthened the reliability of our entire modeling pipeline. The variable vitamin_d_level appeared in three out of the four top 10 feature lists, and vitamin_d_supplement was picked up by RFE. Together, these results suggest that vitamin D status holds statistical importance and biological relevance when predicting lymph node metastasis.

Clinically, vitamin D-related features’ strong and consistent performance across all four classification models is meaningful. Serum vitamin D levels might serve as a prognostic biomarker for lymph node metastasis and as a modifiable risk factor. These findings show that vitamin D supplementation could become an important strategy in managing cervical cancer, especially for patients facing a higher risk of metastatic spread.

**Figure 5:**
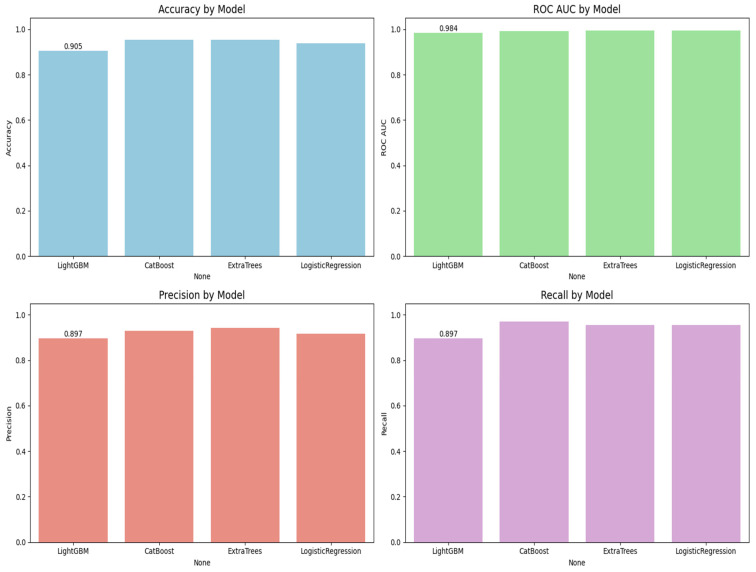
Visualization of the classification report.

Figure 5 is the visualization of the classification report described in Table 3. In this research, we experimentally applied four machine learning models, LightGBM, CatBoost, Extra Trees, and logistic regression, to predict lymph node metastasis. The models were evaluated based on a comprehensive range of metrics, including accuracy, ROC AUC, precision, recall, and classification report for both the positive and negative classes of presence versus absence of metastasis.

**Figure 6:**
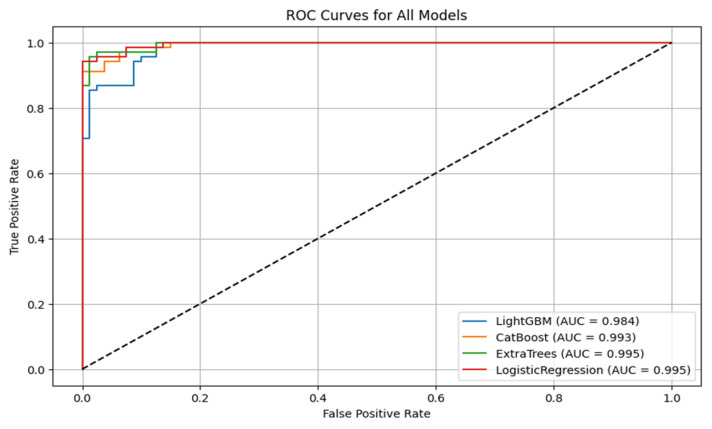
ROC curves for all models.

Figure 6 shows the ROC curves for all the models. CatBoost and Extra Trees performed the best overall, producing an accuracy of 95.27%. CatBoost provided an ROC AUC of 0.9930, a precision of 0.9296, and a recall of 0.9706, and this implies a significant trade-off between the ability to detect metastatic cases while still providing high predictive confidence correctly. Extra Trees performed marginally better than CatBoost in ROC AUC (0.9946) and obtained higher precision (0.9420) but lower recall (0.9559) than CatBoost. This indicates that while both models are likely very sensitive, Extra Trees may be marginally more proficient at correctly predicting non-metastatic cases without losing significant ground in sensitivity.

**Figure 7:**
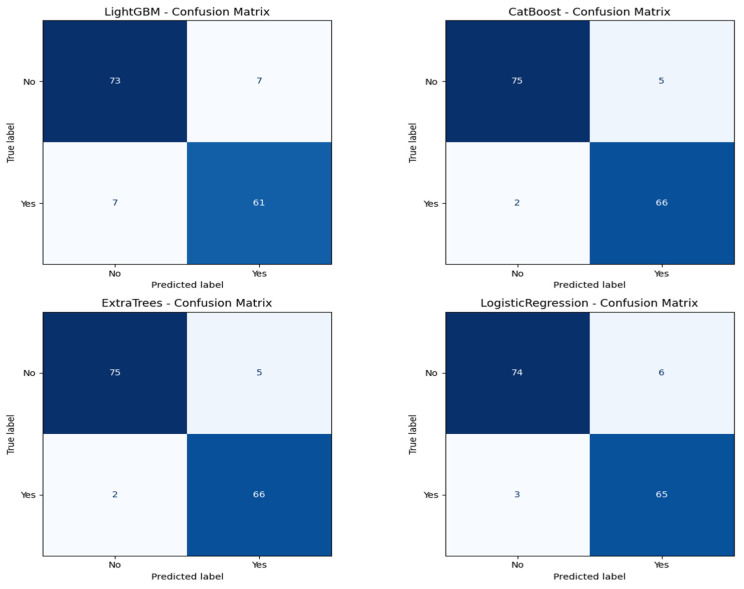
Confusion matrix of classification models.

Algorithm 1 is the mutual information based catboost feature selection and classification. Logistic regression has an excellent performance with an accuracy of 93.92%, an ROC AUC of 0.9954, a precision of 0.9155, and a recall of 0.9559. Its ROC AUC was exceptionally high and better than those from any other models, indicating excellent discrimination between metastatic and non-metastatic classes. However, its lower precision than Extra Trees suggests a small positive rate. Logistic regression achieved a good trade-off between sensitivity and specificity, and therefore, it is a helpful model to set the baseline for clinical interpretation due to its natural simplicity. LightGBM with a higher training time achieved high accuracy (90.54%), ROC AUC (0.9844), precision (0.8971), and recall (0.8971) and was scored the lowest in comparison with the rest. It did well overall but finished closely behind CatBoost, Extra Trees, and logistic regression on all major scores. The lower recall than CatBoost and Extra Trees implies that LightGBM failed to seize a larger number of metastatic cases, which is very dangerous in real clinical use. The classification reports also showed that CatBoost performed best in terms of F1-score for metastatic cases (Class 1) and had the best one-harmonic average of precision and recall of any models. This indicates that CatBoost is strong in correctly predicting positive cases and decreasing the number of false optimistic predictions. Extra Trees came close behind with an excellent F1-score and could be a great contender, especially if you want to lower the false positive predictions more. Figure 7 shows the confusion matrix for all the classification models.
**Algorithm 1.** Mutual information-based CatBoost attribute selection and classification  CatBoost attributes were selected and classified [49] well based on mutual information, and the model performed well, with high accuracy in this combination.  **Step 1**: Data preparation.    $S={\left\{\left(y^{\left(k\right)},z^{\left(l\right)}\right)\right\}}_{k=1\;}^{\;N}$(25)  In the full dataset with N samples, each data point consists of    $y^{\left(k\right)}=[y_{1}^{\left(k\right)},y_{2}^{\left(k\right)}\ldots y_{d}^{\left(k\right)}]\in R^{d}$(26)  Feature vector of sample k contains d.    $y^{\left(k\right)}\in Y\;(Target\;class)$(27)  **Step 2**: $y_{l}\in Y$ computes its mutual information with target Z.    $MI\left(y_{l},Z\right)=\sum_{y_{k\in Y_{k}}}\sum_{z\in Z}P\left(y_{k},z\right)*log(\frac{P\left(y_{k},z\right)}{P\left(y_{k}\right)\cdot P\left(z\right)})$(28)        $y_{k}$—k th feature in the cervical cancer dataset        $Y_{k}$—Set of all possible values that feature $y_{k}$ can take        z—Target variable, Z—Set of all possible target value  **Step 3**: Feature Ranking and Selection.    Rank features by their mutual information score.      $Rank\left(y_{k}\right)=\arg\;sort(MI(y_{k,}z))$(29)    The top K features are selected.      $Y_{selected}=\left\{y_{k}|k\in Top\;K\;Indices\left(MI\left(y_{k},z\right)\right)\right\}$(30)  **Step 4**: Model training with CatBoost.    Train a CatBoost classifier on selected features.      $f\left(Y_{train}^{i}\right)=catboost\;classifier.fit(Y_{train}^{i},z)$(31)      $Y_{train}^{i}$—Training data matrix consist of only top i selected feature      z—target vector

We aimed to correlate serum vitamin D levels from a clinical dataset with gene expression changes observed in calcitriol-treated CaSki cells from the GSE267715 study, focusing primarily on long non-coding RNAs (lncRNAs). By preventing essential procedures for tumor growth, calcitriol has a strong anticancer property in the recurrence cervical cancer model, highlighting the need to preserve an adequate nutritional intake of vitamin D [48]. The calcitriol treatment of CaSki cervical cancer cells led to significant suppression of many oncogenic pathways. Transcriptomic profiling data (GSE267715) from calcitriol-treated CaSki cells is analyzed to identify genes associated with vitamin D-responsive and cervical cancer. Patients with sufficient vitamin D showed lower recurrence and longer DFS. The transcriptomic analysis revealed a strong upregulation of CYP24A1 in which FC is 216.92 and FDR is 0.0093 and a downregulation of high-risk lncRNAs, including RP11-396F22.1, previously linked to poor prognosis in early-stage cervical cancer [50]. These findings show that vitamin D may suppress cervical cancer recurrence by modulating gene expression patterns, particularly through lncRNAs. Vitamin D may serve as a prognostic biomarker and therapeutic adjunct in cervical cancer management. Both clinical and transcriptomic evidence converge to support the anti-tumor potential of maintaining sufficient vitamin D levels in cervical cancer patients. Vitamin D correlates with lower recurrence risk clinically and mechanistically suppresses oncogenic long non-coding RNAs at the molecular level, offering a novel target for therapeutic intervention.

## 4. Results

### 4.1. Clinical Correlation of Vitamin D with Recurrence

This study compares vitamin D levels between patients with and without cervical cancer recurrence. The descriptive statistics in Table 4 show that those without recurrence (recurrence status = 0) had a mean vitamin D level of 24.94 ng/mL and a median of 25.04 ng/mL, with a standard deviation of 7.83 across 638 individuals. Patients who experienced recurrence (recurrence status = 1) had lower Vitamin D levels, with a mean of 14.73 ng/mL, a median of 15.02 ng/mL, and a similar standard deviation of 7.78 observed among 100 individuals.

**Figure 8:**
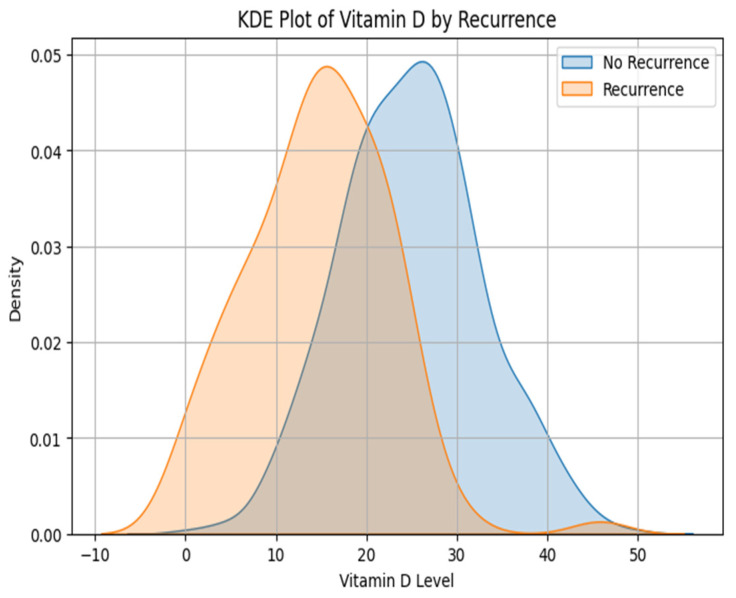
KDE plot of vitamin D by recurrence.

Parametric and non-parametric approaches evaluate whether these differences were statistically significant. Table 5 shows the T-test and Mann–Whitney U test interpretations. The independent samples t-test produced a t-value of 12.186 with a *p*-value below 0.0001, strongly indicating a significant difference under the normality assumption. Similarly, the Mann–Whitney U test, which does not rely on normal distribution assumptions, yielded a U-statistic of 52,878.0 with a *p*-value less than 0.0001. The findings from both tests highlight a significant disparity in vitamin D levels in the groups. Figure 8 is the Kernel Density Estimation (KDE) plot of vitamin D by recurrence. These results suggest a link between Vitamin D deficiency and the risk of cervical cancer recurrence, thus necessitating further exploration into vitamin D’s role as a prognostic marker in managing cervical cancer [51].

**Figure 9:**
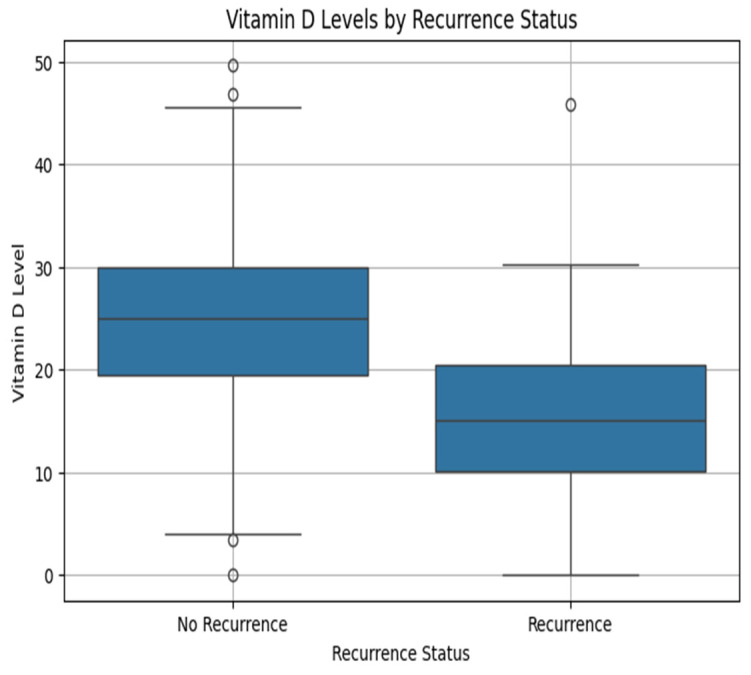
Box plot of vitamin D level by recurrence status.

Figure 9 is the box plot of vitamin D levels by recurrence status, which shows that patients without recurrence have higher vitamin D levels than those with recurrence. It shows the guarding role of vitamin D in cervical cancer recurrence.

**Figure 10:**
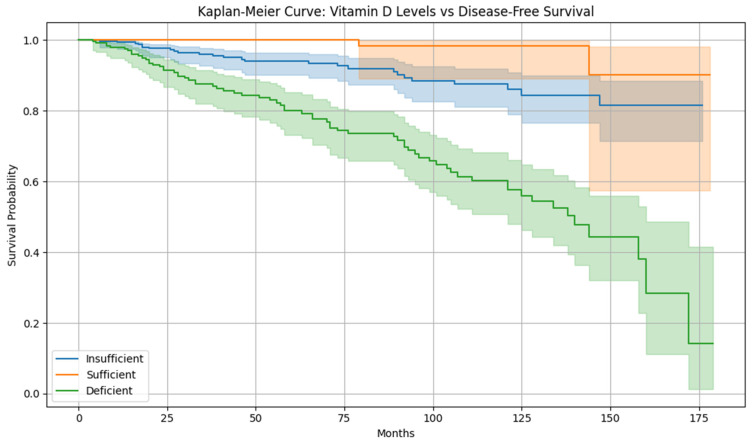
Kaplan–Meier curve for disease-free survival.

Figure 10 is the Kaplan–Meier analysis, which shows that patients with sufficient vitamin D levels have significantly longer disease-free survival when compared with those who are deficient. Furthermore, the Log-rank *p*-value (deficient vs. sufficient) is 1.560274424224108 × 10^−10^. This supports the hypothesis that vitamin D is protective against cervical cancer recurrence.

### 4.2. Key Findings from the Dataset of GSE267715 That Strongly Support the Findings

The integration of transcriptome data from GSE267715 reveals differential regulation of multiple components of vitamin D pathway, including CYP24A1, CYP27B1, VDR, cervical cancer-associated lncRNAs, RP11-396F22, AC017020.2, and CRAT family, which are shown in Table 6. CYP24A1 was strongly upregulated with a fold change of 216.9, and FDR is 0.0093, confirming a robust calcitriol response. These findings support the hypothesis that vitamin D suppresses recurrence by modulating transcriptomic pathways involving vitamin D metabolism and cervical oncogenesis. Genes responsive to calcitriol treatment in CaSki cervical cancer cells from GSE267715, notable vitamin D pathway components and cervical cancer–associated lncRNAs, are differentially expressed, supporting a transcriptional mechanism by which vitamin D may suppress recurrence.

**Figure 11:**
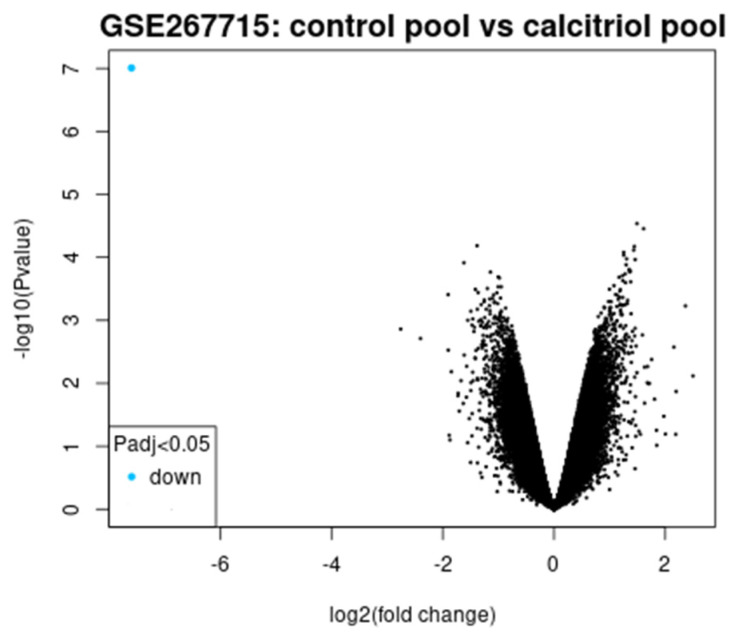
Volcano plot (fold change vs. *p*-value).

Figure 11 shows the volcano plot, which shows the log2 fold change versus log10 (*p*-value). Genes with significant differential expressions that have adjusted *p*-value < 0.05 appear highlighted in blue and are downregulated. The global gene expression changes induced by vitamin D (calcitriol) treatment are also shown.

**Figure 12:**
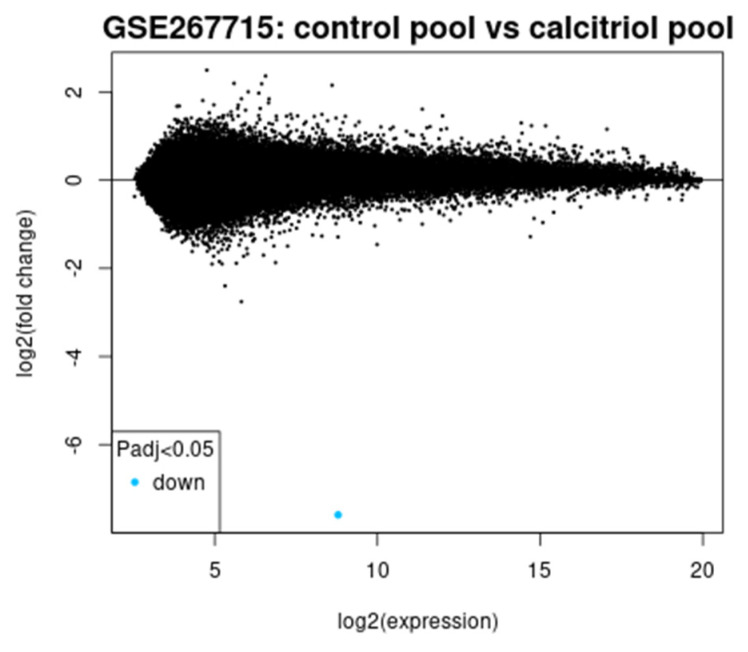
Mean difference plot (mean expression vs. fold change).

Figure 12 is the mean difference plot. The x-axis shows the average expression of the log2 scale, and the y-axis shows the log2 fold change. Points far from zero (y-axis) are genes with large expression changes. Most genes cluster around no change (fold change ≈ 0). The blue point again marks a downregulated significant gene. It confirms that the major expression shifts are rare and targeted, supporting specific suppression effects of calcitriol rather than global shifts.

**Figure 13:**
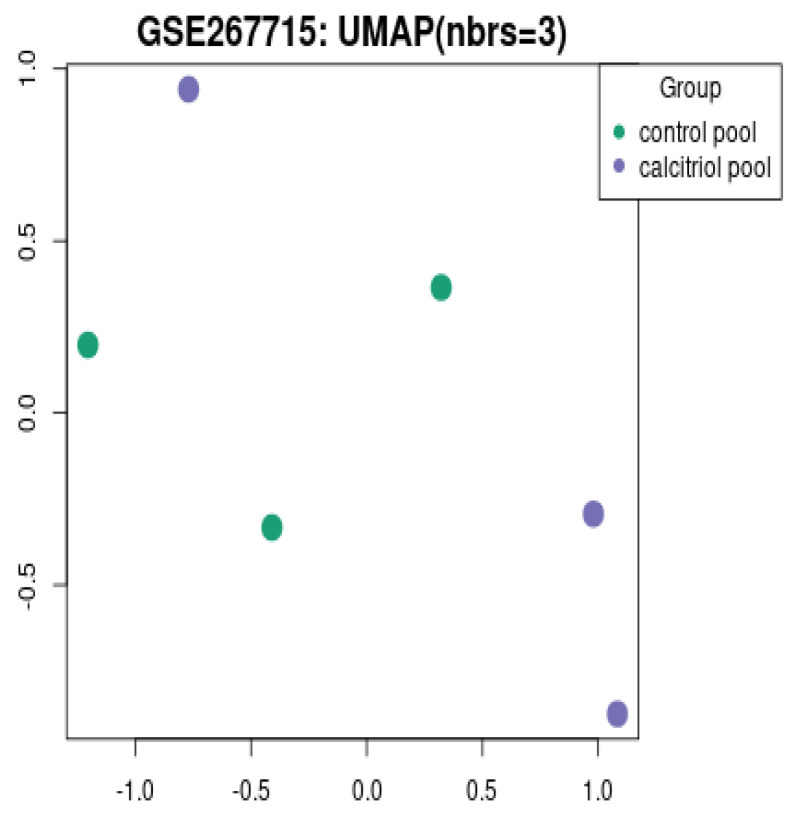
UMAP plot (sample clustering).

Figure 13 shows the Uniform Manifold Approximation and Projection (UMAP), which reduces dimensionality to 2D, clustering samples based on expression profiles. The green dots are the control pool, and the purple dots are the calcitriol-treated pool. There is a clear separation between the vehicle and calcitriol groups. Calcitriol induces distinct transcriptomic profiles in CaSki cervical cancer cells. This supports the hypothesis that vitamin D treatment creates major transcriptomic shifts, contributing to anticancer effects.

**Figure 14:**
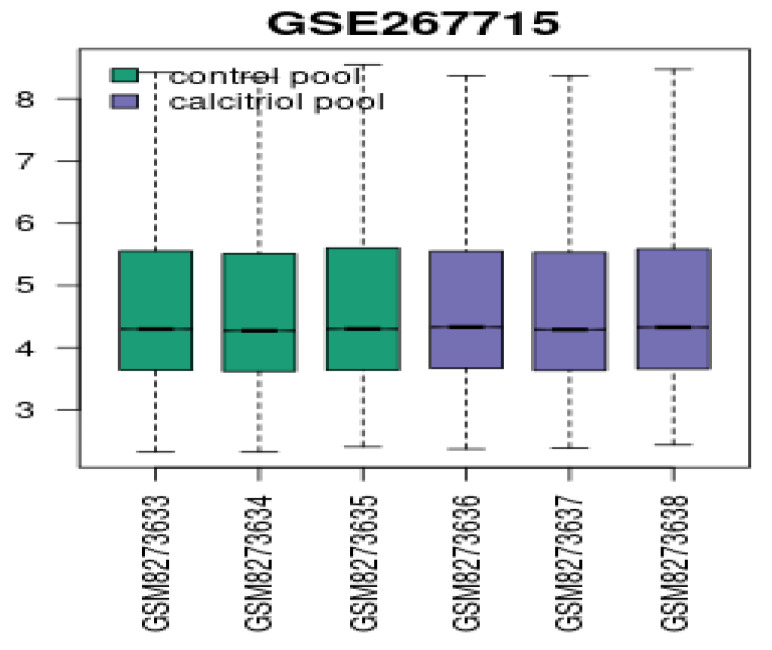
Boxplot of the expression distribution across samples.

Figure 14 shows a boxplot of the overall gene expression levels across samples (GSM IDs). Green is the vehicle control, and purple is the calcitriol-treated group. The expression distributions are comparable, indicating no global biases like RNA degradation or batch effects.

**Figure 15:**
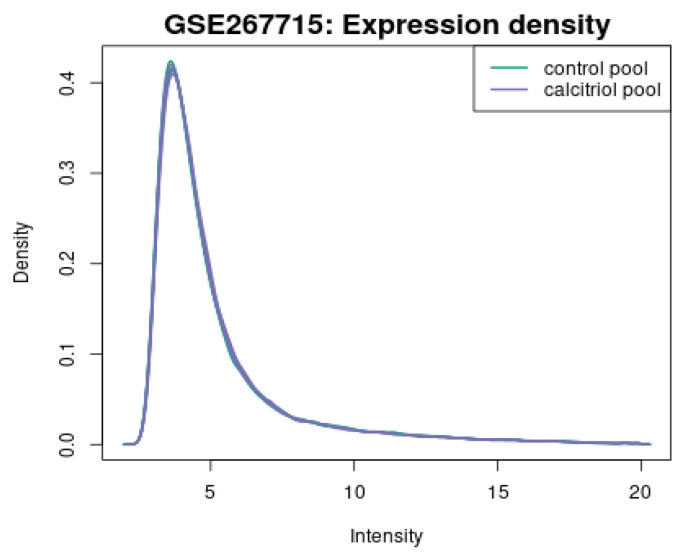
Density plot of expression intensity.

Figure 15 shows the density curves of the overall expression levels and compares the vehicle (green) and calcitriol-treated (purple) groups, with almost identical curves, confirming reasonable quality control.

**Figure 16:**
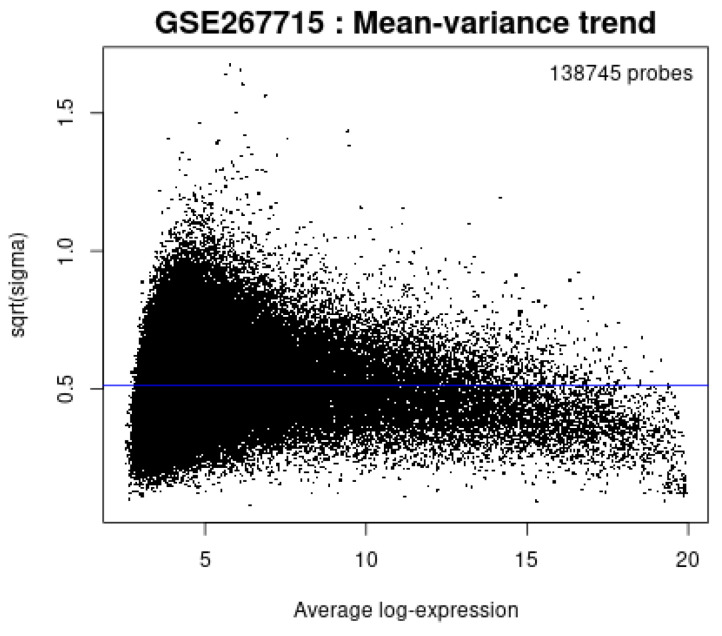
Mean variance plot.

Figure 16 shows the relationship between mean expression level and variance across probes. Variance stabilizes at higher expression, as expected. The blue line shows the threshold or variance trend.

The gene expression data for GSE267715 from theh NCBI GEO database was analyzed, explicitly focusing on genes classified as non-coding to identify long non-coding RNAs (lncRNAs) and, after isolating the lncRNAs, ranked based on the absolute magnitude of their fold changes, selecting the top ten most differentially expressed lncRNAs following calcitriol treatment in CaSki cervical cancer cells. Many of the lncRNAs detected by the microarray were unnamed because they represent transcripts identified at the genomic level but not fully characterized by official gene symbols in standard databases like GENCODE or LNCipedia. RP11-396F22.1 was a named and biologically validated lncRNA recognized in prior studies for its role. Mainly, RP11-396F22.1, which had been linked to an early-stage cervical cancer poor prognosis, was downregulated following calcitriol therapy [52]. This dual approach data-driven selection based on differential expression combined with literature-supported biological relevance allowed us to prioritize RP11-396F22.1 among many unnamed lncRNAs.

**Figure 17:**
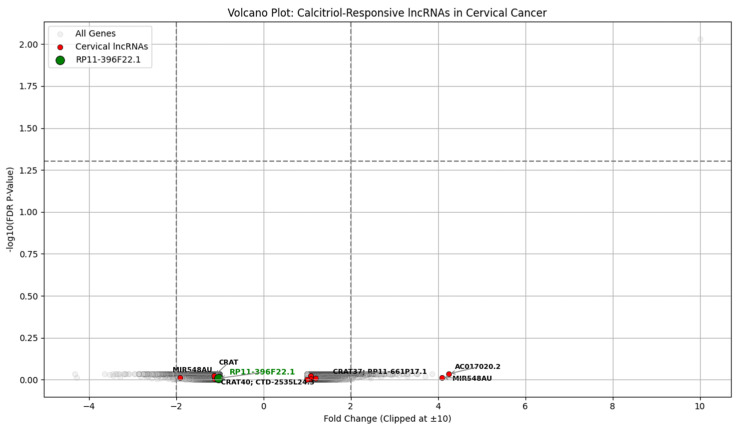
Volcano plot of calcitriol responsive lncRNA in cervical cancer.

The top cervical cancer-related long non-coding RNA is shown in Table 7. The transcriptomic profiling of GSE267715 shows several lncRNAs associated with cervical cancer modulated by calcitriol treatment, including AC017020.2, which is 4.25-fold, and CRAT37, which is 1.20-unfold. Figure 17 is the calcitriol-responsive lncRNA in the cervical cancer volcano plot. Although FDR-adjusted *p*-values were insignificant, these genes are biologically relevant and support the hypothesis that vitamin D modulates the expression of non-coding transcripts implicated in recurrence and tumor progression.

## 5. Discussion

The integrated clinical and bioinformatics analysis shows a link between vitamin D sufficiency and reduced cervical cancer recurrence risk. Clinically, patients with vitamin D levels above 30 ng/mL show improved disease-free survival, while vitamin D-deficient patients are at high risk of recurrence. This relates to immunological evidence that vitamin D modulates tumor immune evasion and cellular differentiation. The research does not claim that vitamin D supplementation alone will reduce cervical cancer recurrence. It reveals a significant association between higher vitamin D levels and lower recurrence risk and suggests that vitamin D influences recurrence-related gene expression, including long non-coding RNA. These findings support that vitamin D is a regulatory compound within a therapeutic framework.

The transcriptomic analysis of calcitriol-treated CaSki cervical cancer cells (GSE267715) supports this clinical observation at a molecular level. CYP24A1, a key regulator of vitamin D metabolism, is upregulated with FC of 216.92 and FDR of 0.0093, confirming the activation of the vitamin D signaling pathway. The differential expression of several lncRNAs involved in cervical cancer progressions, like RP11-396F22.1, AC017020.2, and MIR548AU.RP11-396F22.1, identified as a poor prognosis marker in early-stage cervical cancer [53], was modestly downregulated following calcitriol exposure. This suggests that vitamin D relieves the recurrence risk by regulating lncRNAs involved in oncogenic signaling and immune modulation. The gene expression data derived from in vitro models and simulated expression were used for exploratory clinical modeling. The consistency between clinical and transcriptomic findings strengthens the findings. Future studies should validate these lncRNA markers in patient samples and assess the therapeutic utility of vitamin D supplementation.

## 6. Conclusions

This research integrates clinical and molecular data to offer a novel mechanistic pathway linking vitamin D levels to cervical cancer recurrence by modulating long non-coding RNAs. These findings support vitamin D’s role as a supportive agent in managing cervical dysplasia by improving systemic immune readiness and inflammation control. Vitamin D is not a standalone treatment but a promising adjuvant regulatory compound that enhances anti-recurrence mechanisms, possibly through the modulation of lncRNA expression and immune pathways.

The clinical findings established that patients experiencing recurrence have significantly lower vitamin D levels. This observation is supported by transcriptomic evidence from GSE267715, which shows that calcitriol treatment leads to the downregulation of the oncogenic lncRNA in CaSki cervical cancer cells. Advanced feature selection methods and machine learning classifiers in predictive modeling enhance the reliability and interpretability of recurrence risk. Techniques such as ANOVA F-test, mutual information, chi-squared test, and RFE ensured optimal feature subset selection. At the same time, classifiers like LightGBM, CatBoost, logistic regression, and Extra Trees show high predictive accuracy. In conclusion, vitamin D holds potential not only as a biomarker but also as a modifiable therapeutic adjunct in managing cervical cancer recurrence and increasing disease-free survival. Although direct studies in cervical cancer are limited, its suppression by vitamin D and known oncogenic mechanisms support its potential role in cervical cancer recurrence.

## Figures and Tables

**Table 1:** Clinical representation of important features.

Features	Patients Count	Mean	Std. Dev.	Min	25%	Median	75%	Max
vitamin_d_level	738	24.84	7.92	5.10	19.90	24.95	29.80	51.30
Duration of Symptoms	738	1.87	0.75	1	1	2	2	3
FIGO Stage	738	4.26	1.94	1	3	4	5	8
Lymph Node Metastasis	738	0.461	0.499	0	0	0	1	1
Recurrence Status	738	0.135	0.342	0	0	0	0	1

**Table 2:** Feature importance score for clinical prediction (LightGBM output).

Feature	Importance Score
vitamin_d_level	0.30
vitamin_d_supplement_Yes	0.25
FIGO_STAGE	0.20
Symptoms	0.15

**Table 3:** Different models’ classification reports.

Model	Accuracy	ROC AUC	Precision	Recall
LightGBM	0.9054	0.9844	0.8971	0.8971
CatBoost	0.9527	0.9930	0.9296	0.9706
ExtraTrees	0.9527	0.9946	0.9420	0.9559
Logistic Regression	0.9392	0.9954	0.9155	0.9559

**Table 4:** Descriptive statistics of recurrence status vs. vitamin D.

Recurrence Status	Mean Vitamin D (ng/mL)	Median Vitamin D (ng/mL)	Std. Dev.	Sample Size (*n*)
0 (No Recurrence)	24.94	25.04	7.83	638
1 (Recurrence)	14.73	15.02	7.78	100

**Table 5:** T-test and Mann–Whitney U test interpretations.

Test	Statistic	*p*-Value	Interpretation
T-test	t = 12.186	<0.0001	Significant difference (parametric)
Mann–Whitney U test	U = 52878.0	<0.0001	Significant difference (non-parametric)

**Table 6:** Vitamin D pathway components and cervical cancer–associated lncRNAs.

Gene Symbol	Fold Change	FDR	Relevance
CYP24A1	216.92	0.0093	Vitamin D target (strongly upregulated)
CYP27B1	1.04	0.9900	Enzyme that activates vitamin D
VDR	−1.03	0.9625	Vitamin D receptor
GC	1.05	0.9995	Vitamin D-binding protein
RP11-396F22.1	−1.03	0.9821	Cervical cancer poor prognosis lncRNA
AC017020.2	+4.25	0.9239	Immune-related lncRNA
MIR548AU	−1.92	~0.97	Immune/cervical-related lncRNA
CRAT8/CRAT37/CRAT40	~1.08 to −1.12	~0.95–0.98	Cervical cancer-associated transcripts
RP11-89H19.1	+1.08	0.9873	Antisense to VDR gene

**Table 7:** Top cervical cancer-related lncRNA.

Gene Symbol	Fold Change	FDR *p*-Value
AC017020.2	4.25	0.9239
MIR548AU	4.09	0.9691
MIR548AU	−1.92	0.9689
CRAT37; RP11-661P17.1	1.20	0.9782
CRAT	−1.13	0.9499
CRAT40; CTD-2535L24.3	−1.12	0.9877

## Data Availability

The datasets used and analyzed during the current study were collected from Shanmuga Hospital Salem. A publicly accessible dataset from NCBI GEO was downloaded from GSE267715.
